# Metabolomic Profiling and Biological Activities of *Pleurotus columbinus* Quél. Cultivated on Different Agri-Food Byproducts

**DOI:** 10.3390/antibiotics10101245

**Published:** 2021-10-14

**Authors:** Paola Angelini, Roberto Maria Pellegrino, Bruno Tirillini, Giancarlo Angeles Flores, Husam B. R. Alabed, Federica Ianni, Francesca Blasi, Lina Cossignani, Roberto Venanzoni, Giustino Orlando, Luigi Menghini, Claudio Ferrante

**Affiliations:** 1 Department of Chemistry, Biology and Biotechnology, University of Perugia, 06100 Perugia, Italy; paola.angelini@unipg.it (P.A.); roberto.pellegrino@unipg.it (R.M.P.); giancarlo.angelesflores@studenti.unipg.it (G.A.F.); husambr.alabed@studenti.unipg.it (H.B.R.A.); roberto.venanzoni@unipg.it (R.V.); 2 Department of Biomolecular Sciences, University of Urbino, 61029 Urbino, Italy; bruno.tirillini@uniurb.it; 3 Department of Pharmaceutical Sciences, University of Perugia, 06126 Perugia, Italy; federica.ianni@unipg.it (F.I.); francesca.blasi@unipg.it (F.B.); lina.cossignani@unipg.it (L.C.); 4 Department of Pharmacy, Università degli Studi “Gabriele d’Annunzio”, Via dei Vestini 31, 66100 Chieti, Italy; giustino.orlando@unich.it (G.O.); claudio.ferrante@unich.it (C.F.)

**Keywords:** *Pleurotus columbinus*, metabolomics, phenolic compounds, antimicrobial properties

## Abstract

The genus *Pleurotus* (Fr.) P. Kumm (Pleurotaceae, Basidiomycota) comprises a cosmopolitan group of mushrooms highly appreciated for their nutritional value and health-promoting benefits. Despite there being many studies about the phytochemical composition of *Pleurotus* spp., there are very few reports dealing with the phytochemistry, antioxidant and antimicrobial activities of *P. columbinus* Quél. In this study, a mass spectrometry ultra-performance liquid chromatography mass spectrometry (UHPLC)-QTOF method, coupled with principal component analysis (PCA), was applied to the *P. columbinus* metabolome in order to investigate the influence of different agri-food residues as growth substrates for *P. columbinus* cultivation, on the bioactive chemical profile of fruiting bodies and evaluated their potential as antioxidants and antimicrobials. Additionally, a quantitative HPLC-DAD-MS analysis was conducted on phenolic and flavonoid compounds, that could explain, albeit partially, the observed biological effects of *P. columbinus* extracts. The qualitative metabolic profile identified 97 metabolites, whereas the quantitative HPLC-DAD-MS analysis confirmed the presence of phenolic and flavonoids, in the mushroom extracts, which also showed intrinsic scavenging/reducing and antimicrobial effects. The antibacterial effects were particularly evident against *Escherichia coli*, whereas *Tricophyton* and *Aspergillus* were the dermatophytes more sensitive to the mushroom extracts. The present study supports more in-depth investigations, aimed at evaluating the influence of growth substrate on *P. columbinus* antimicrobial and antioxidant properties. The extracts from *P. columbinus* revealed valuable sources of primary and secondary metabolites, thus suggesting potential applications in the formulation of food supplements with biological properties, above all in terms of antioxidant and antimicrobial properties.

## 1. Introduction

The genus *Pleurotus* (Fr.) P. Kumm (Pleurotaceae, Basidiomycota) comprises a cosmopolitan group of mushrooms highly appreciated for their nutritional value and health-promoting benefits [1,2]. *Pleurotus* spp., commonly known as oyster mushrooms, are classified as white-rot fungi and after *Agaricus bisporus* (J.E. Lange) Imbach and *Lentinula edodes* (Berk.) Pegler, they represent a very diffuse group of cultivated edible mushrooms worldwide [3].

At present, *Pleurotus* spp., including *P. citrinopileatus* Singer, *P. djamor* (Rumph. ex Fr.) Boedijn, *P. eryngii* (DC.) Quél, *P. flabellatus* Sacc, *P. florida* Singer and *P. ostreatus* (Jacq.) P. Kumm., have achieved wide acclaim as nutraceuticals due to their exceptional nutritional and medicinal properties and their ability to grow on various sources of agricultural waste [4,5].

Nutritionally, oyster mushrooms are low-fat, low-energy, low sodium and cholesterol-free food items. In addition, they are valued as an important source of water-soluble vitamins, proteins, minerals, chitin and glucans (functional polysaccharides). Furthermore, *Pleurotus* spp. contain biologically active compounds such as ergosterol (provitamin D2), phenolic acids, antioxidant amino acid, ergothioneine and lovastatin [6].

The fruiting bodies and mycelia of several *Pleurotus* species possess various biological activities, such as anti-inflammatory, immune-stimulating and immune-modulating, antitumor, anticancer, ribonuclease activity, hypolipidemic, antimicrobial and antioxidant [2,7,8,9,10,11,12,13,14,15,16].

Chemical analyses have shown that many of the biologically active compounds isolated from *Pleurotus* mushrooms belong to hemicelluloses, polysaccharides, lipopolysaccharides, peptides, proteins, glycoproteins, nucleosides, triterpenoids, complex starches, lectins and lipids [17].

*Pleurotus* spp. also have high ability to use a wide variety of lignocellulosic waste such as sawdust (i.e., *Populus* spp., *Quercus* spp., *Fagus sylvatica* L.), rice straw (*Oryza sativa* L.), wheat straw (*Triticum aestivum* L.), corn stover (*Zea mays* L.), grass residues [*Cynodon dactylon* L. Pers.], sunflower residues (*Helianthus annuus* L.), grape marc (*Vitis vinifera* L.), olive mills (*Olea europaea* L.), banana straw (*Musa x paradisiaca* L.), date-palm leaves (*Phoenix dactylifera* L.), hazelnut leaves (*Corylus* spp.), coffee husks, etc. [18,19,20,21].

After mushroom harvesting, the residual substrate can be used as a type of bio-fertilizer or an animal feeding, but also for enzymes production [22]. Therefore, the cultivation of mushroom meets the needs of current sustainable agriculture, while at the same time supplying a functional food [23]. In this regard, it is sensitive to note that different agricultural byproducts have been employed as substrates for cultivating *Pleurotus* mushrooms, namely, banana leaves, peanut hull, corn leaves and others [24]. The most cultivated *Pleurotus* species included on residual substrates are *P. ostreatus*, *P. sajor-caju*, *P. eous* and *P. florida* [24].

Extensive research regarding the influence of substrate formulations used for mushrooms cultivation on the bioactive chemical profile of different species of *Pleurotus* has been investigated by researchers [3,25,26,27,28,29]. However, it should be also considered the influence of post-harvesting conditions which could lead to a loss of biomolecules, especially phenolic compounds, thus leading to discoloration of the mushroom [24].

Cereal straws represent a by-product of cereal threshing and are produced in large volume. Chopped wheat straw is one of the main components of the growth medium used for the cultivation of *Pleurotus* spp., but often, in order to increase the yield, it is supplemented with materials rich in proteins [3]. Kinge et al. [28] found that *P. ostreatus* grown on sawdust is characterized by better growth and nutritional properties than that grown on corn cobs. The analysis of the bioactive components revealed the presence of flavonoids, polyphenols, saponins, triterpenoids and steroids. Within the same species of *Pleurotus*, the protein content would appear to be that mushroom nutrient most influenced by the type of growth substrate, followed by crude fiber and carbohydrates [29].

As *P. ostreatus* is the most common species considered in the literature, and apparently the best known, this name has often been used indiscriminately and it has been confused with other species, such as *P. columbinus* Quél., *P. pulmonarius* (Fr.) Quél., etc. [30].

**Figure 1:**
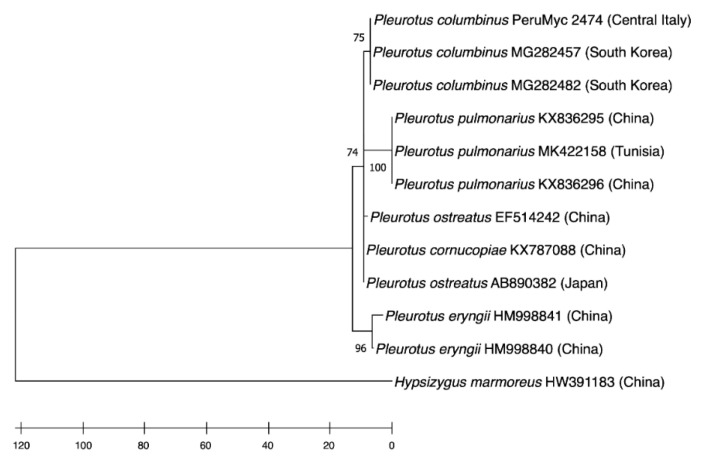
Maximum parsimony tree (obtained using the Min-mini heuristic algorithm) showing the phylogenetic position of the *Pleurotus columbinus* strain used in the present study (PeruMyc 2474). Sequences representing different *Pleurotus* spp. were used along with *Hypsizygus marmoreus* (seq. HW391183) as outgroup.

The names *P. columbinus* Quél. has been applied to blue-greenish blue variant of *P. ostreatus*. Hilber [31] considers *P. columbinus* a variety of *P. ostreatus* (*P. ostreatus* var. *columbinus* Quél., Enchir. Fung.: 148. 1886) and demonstrated a high degree of intercompatibility (>85%) between var. *columbinus* and var. *ostreatus*. The investigation of Zervakis and Labarère [32], based on isoenzymes from 23 *Pleurotus* spp. isolates examined by polyacrylamide gel electrophoresis isoelectric and protein blotting, however, demonstrated that *P. columbinus* (Figure 1) could be regarded as a separate taxon.

Despite there being many studies about the phytochemical composition of *Pleurotus* spp., also highlighted by recent reviews [33,34,35] there are very few studies dealing with the phytochemistry, antioxidant and antimicrobial activities of *P. columbinus* Quél [36,37]. Metabolomics is a new discipline which is defined as the monitoring of metabolite concentration in fungi, bacteria and plants. Nowadays, the metabolomics-based approach has been gradually applied in the field of edible and medicinal mushrooms to gain insight into the chemical compositions of biological processes and the understanding response of mushrooms to certain environmental conditions [38].

In this study, a mass spectrometry ultra-performance liquid chromatography mass spectrometry (UHPLC)-QTOF method, coupled with principal component analysis (PCA), was applied to the *P. columbinus* metabolome in order to investigate the influence of different agri-food residues as growth substrates for *P. columbinus* cultivation, on the bioactive chemical profile of fruiting bodies and evaluated their potential as antioxidants and antimicrobials. Additionally, a quantitative HPLC-DAD-MS analysis was conducted on phenolic and flavonoid compounds, possibly involved in the observed biological effects of *P. columbinus* extracts.

## 2. Results and Discussion

### 2.1. Mushroom Identification

The morphological characteristics of *Pleurotus columbinus* fruiting body correspond to those reported by Bas et al. [39].

Considering the influence of cultivation conditions on morphological and physiological features, the DNA barcoding is requested for the identification of *Pleurotus* species. The exact characterization and identification of medicinal mushrooms is fundamental for exploiting their full potential in food and pharmaceutical industries [29].

The taxonomic affiliation of the mushroom strain was performed via targeting the internal transcribed region of the ribosomal DNA. The ITS sequence of sample PeruMyc 2474 was consistent with the species *P. columbinus*; accordingly, a phylogenetic tree with *Hypsizygus marmoreus* as an outgroup (Figure 1).

The PeruMyc 2474 strain clearly clustered with other *P. columbinus* strains and were related to *P. pulmonarius*, *P. ostreatus* and *P. eryngii,* as well.

### 2.2. Untargeted LC-MS/MS-Based Metabolomics

**Figure 2:**
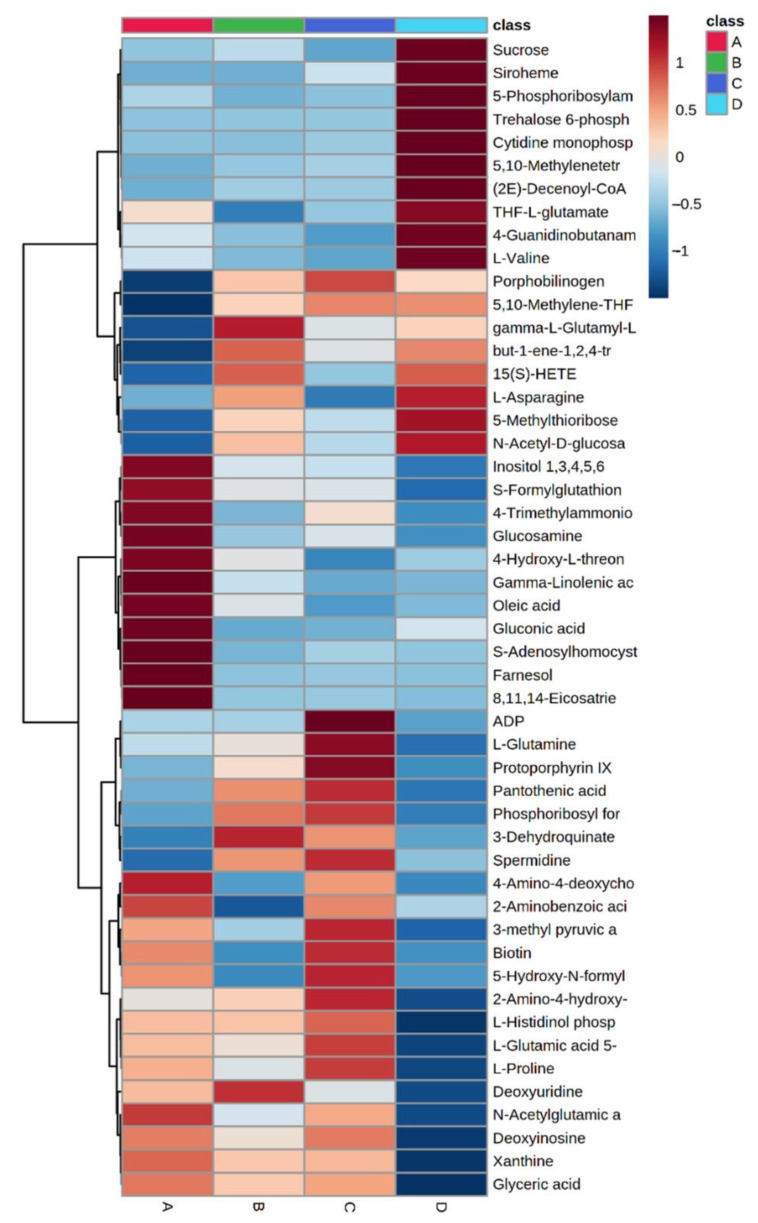
Heatmap using of the 50 most significant metabolites from the ANOVA test. In the heatmap columns, red color indicates higher relative levels of metabolites, whereas the blue color suggests a minor content of them.

**Figure 3:**
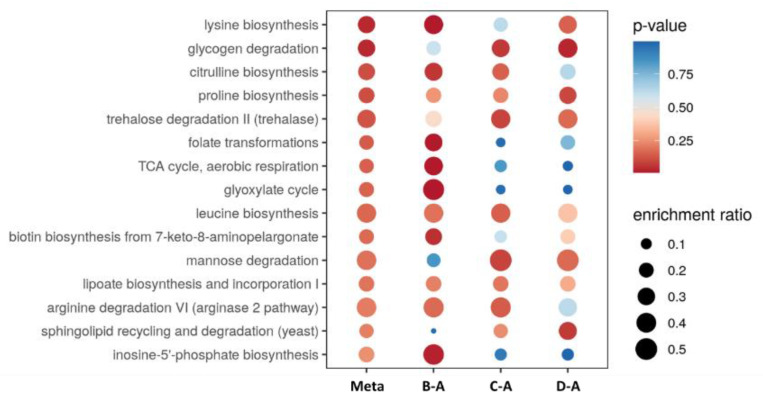
Metabolic profiles of the fungus grown in substrates B–D with respect to substrate A taken as reference. In the figure, red color indicates higher probability of metabolic pathway activation, whereas the blue color suggests a minor one.

In this study, the chemical profile of *P. columbinus* was evaluated through mass spectrometry ultra-performance liquid chromatography mass spectrometry (UHPLC)-QTOF method, coupled with principal component analysis (PCA). The full list of metabolites annotated using the mummichog algorithm is included in Table S1 (Supplementary Material). The data matrix showing the annotation and peak areas for each sample was subjected to statistical analysis. The ANOVA performed with a *p*-value cut-off of 0.001 found 97 significant and 136 non-significant metabolites. In Table S2 (Supplementary Material), it is reported the list of significance using post hoc analysis (Fisher’s LSD), whereas Figure 2 shows the heatmap using the 50 most significant metabolites from the ANOVA test. Specifically, the fungi grown in the substrate D showed higher levels of carbohydrates, such as sucrose and mannose, compared to the reference substrate A, whereas the substrate B is related to higher amounts of aminoacids, such as L-glutamine and L-proline, in the fruiting bodies. This is also consistent with the functional metanalysis carried out to contextualize the metabolomics profile (Figure 3). Comparisons were made between the metabolic profiles of the fungus grown in substrates B–D with respect to substrate A taken as reference. The most evident thing is that the metabolic pathways are strongly influenced by the chemical composition of the growth substrate. Some differences can be tentatively explained. For example, mannose degradation is greater for the fungus grown on substrate C. Indeed, this substrate contains, among other things, coffee grounds which are rich in mannose. A similar trend is noted for the metabolic pathway of leucine biosynthesis which is increased for the fungus grown on substrate C. In this substrate, free leucine is scarce compared to substrates B and D where the presence of soybeans constitutes an immediate source of free leucine.

### 2.3. Phenolic and Flavonoid Determination via HPLC-DAD-MS

An HPLC-DAD-MS analysis was also carried out in order to measure the levels of selected phenolic and flavonoid compounds, namely, gallic acid, hydroxytyrosol, catechin, chlorogenic acid, epicatechin and benzoic acid, that play a major role in the antioxidant/antimicrobial response following mushroom extract administration [40,41]. Specifically, Table 1 shows that the level of gallic acid is higher in extracts A and C, whereas extract B do not show a relevant amount of this compound. In the extracts B–D, the catechin fraction was present at higher concentrations, compared to extract A. This last extract also had the highest epicatechin content, whereas extract A showed higher benzoic acid concentration. According to the quantitative analysis conducted, extract C was the richest, in terms of qualitative and quantitative composition in phenolic compounds.

### 2.4. Antimicrobial and Antioxidant Effects

The antimicrobial activity of the extracts A–D are shown in Table 2, also in comparison with reference antimicrobial drugs ciprofloxacin, fluconazole and griseofulvin. All extracts from mushroom displayed antimicrobial activity in the concentration range of 6.25 to 200 μg/mL. Regarding the yeasts, *C. parapsilosis* (YEPGA 6551) was the most sensitive strain to the extracts, with MIC ranges of 31.49- >200 µg mL^−1^, while *C. albicans* (YEPGA 6379) showed the least sensitivity to the mushroom extracts. The results of the growth inhibition of yeast strains evidenced a major activity of the extract A, underlining the role of growth substrate on *P. columbinus* extract properties. With reference to bacteria, the strongest inhibition was observed for the extracts B and C [MIC 6.25–12.5 μg/mL against *E. coli* (ATCC 10536) and PeryMycA 2]. Collectively, Gram− bacterial strains (PeruMyc 2, 3, 5 and 7) were less sensitive to mushroom extracts than that of Gram+ ones. Intriguingly, the *B. cereus* strain PeruMycA 4 showed the lowest MIC values. All results from the tested extracts showed active inhibition of dermatophytes growth. Regarding *A. currey* (CCF 5207), it was the most sensitive fungal species to mushroom extracts, with MIC range between 9.92 and 79.37 μg/mL. Values of MIC < 100 µg/mL was considered as an index of high antimicrobial activity (Dogan et al. 2013). The highest antimicrobial activity of riseofulvin was against *T. tonsurans* (CCF 4834) (MIC: 0.125–0.25 μg/mL). On the other hand, the present data did not permit to whether the isolates were resistant to the griseofulvin as no breakpoints have yet been established.

Regarding the antioxidant activity, experimental data were normalized and expressed as EC_50_ values (µg/mL) for each mushroom extract and Trolox, which was used as reference antioxidant compound. The results were given in Table 3. Values for DPPH radical scavenging activity varied between 2.25 and 4.98; extract B was the most active. Decidedly low was the activity of the extract A, with a mean value of 4.98, whilst the extract C had a medium–low mean value (3.81). Values for ABTS radical scavenging activity varied between 4.34 and 6.16, and the best activity was shown by the extracts B, with a mean value of 4.34 referred to the Trolox. The values for β-carotene-linoleic acid assay varied between 7.47 and 11.65; the higher activity was showed by the extract B, with a mean value of 7.47 referred to the Trolox, while lower antioxidant effects were detected for the other extracts. Although the content of phenolic compounds was previously related to the scavenging/reducing and antimicrobial properties of extracts, especially in the case of polar extracts [42,43], in the present study the intrinsic antioxidant and antimicrobial abilities of the extracts seemed to be dependent, at least in part, from the content of the gallic acid present in the extracts (their correlation coefficients with gallic acid were <0.43). Matrix analysis (Table S3) revealed a strong positive correlation (correlation coefficients > 0.89) of quantitative presence of gallic acid in the extracts and the antioxidant activities. Furthermore, among the three tests used for antioxidant activity a largely positive correlation was observed as expressed by coefficients falling in the range 0.88–0.99. On the other hand, antioxidant properties of the extracts were less affected by the presence of other detected flavonoids and phenolics. This reflected the complexity of the analyzed biological matrices. Regarding the antimicrobial properties, the results from the present study did not provide the optimal substrate for the cultivation of fungi with antimicrobial properties; however, the effect of the substrate was present and should be deeply considered in view of the production of antimicrobial extracts from *Pleurotus* species. For instance, the differences in the metabolic pathway activation induced by the substrates seemed to affect the sensitivity of *Candida* species to the mycostatic effects by the extracts. Future studies need to unravel the mechanism underlying this effect.

## 3. Materials and Methods

### 3.1. Chemical and Reagents

20-Azino-bis-(3-ethylbenzothiazoline-6-sulphonate) diammonium salt (ABTS), 6-hydroxy-2,5,7,8-tetramethyl-2-carboxylic acid (Trolox), 2,2-diphenyl-1-picrylhydrazyl (DPPH), ferric chloride (FeCl3), Mueller–Hinton broth (MHB), Rose Bengal Chloramphenicol Agar (RBCA), Malt Extract Agar (MEA), Tryptic Soy Agar (TSA), Sabouraud Dextrose Agar (SDA), RPMI (Roswell Park Memorial Institute) 1640 medium, purity grade organic solvent (Methanol), ciprofloxacin, fluconazole and griseofulvin, were purchased from Sigma (Sigma-Aldrich GmbH, Hamburg, Germany).

### 3.2. Mushroom Material

The fruiting bodies of the strain of *P. columbinus* (PeruMyc 2474) were collected in April 2019, in Marmore Waterfall (Terni, Umbria, Italy), a Special Area of Conservation and Special Protection Area (SAC/SPA IT5220017) of the Natura 2000 EU-wide network (Habitat Directive 92/43/EEC). Basidiomata identification was carried out by macro and micro-morphological analysis [39,44]. The Vaucher specimens were deposited in the Mycological Herbarium MPeru (ID: 62) at the University of Perugia [Department of Chemistry, Biology and Biotechnology (DCBB), Perugia, Italy].

For the isolation of mycelia, portions (less than 5 mm) of context were excised aseptically from inside the basidiome, transferred into Petri dishes containing Rose Bengal Chloramphenicol agar (Sigma-Aldrich, Milan, Italy) and incubated for 7 d at 24 °C [45,46]. The mycelial strains are deposited in the DCBB culture collection and subcultured on MEA medium every three months.

### 3.3. Molecular Identification

Angelini et al. [47] method was used to extract the total genomic DNA from ten days mycelium grown in MEA. The detailed protocol is included in our recent paper [2]. Whereas the phylogenetic analysis was conducted according to literature [48,49,50].

### 3.4. Spawn Production

*P. columbinus* spawn was produced following the standard method with the use of barley grains. Details about the protocol followed are reported in a previous paper of ours [2].

### 3.5. Mushroom Cultivation Substrates

The *P. columbinus* strains were cultivated on four substrates consisting of (A) wheat straw in 1:1 *w/w* ratio with beech sawdust (as control), (B) wheat straw in 4:2:1 *w*/*w/w* ratio with oak sawdust and soya beans, (C) wheat straw in 4:2:1 *w*/*w/w* ratio with oak sawdust and coffee grounds and (D) wheat straw in 3:2:1 *w*/*w/w* ratio with beech sawdust and soya beans. Wheat straw, sawdust, soya beans and coffee ground were obtained from the “Soc. Coop. Umbria Verde farm” (Perugia, Central Italy).

Soya beans make certain free amino acids such as Glutamate, Aspartate, Leucine, Arginine, Serine, Lysine and Proline readily available. Coffee grounds are particularly rich in sugars such as mannose, galactose and arabinose. The detailed protocol is included in our recent paper [2].

### 3.6. Preparation of Mushrooms Methanol Extracts

The fruiting bodies of *P. columbinus* grown on substrates A–D were manually collected and selected discarding parts presenting non-healthy aspect, of physical damage. Fresh material was grossly divided in slices and immediately macerated in methanol for seven days at 20 °C (1:10 *w*:*v*). Extracts were then centrifuged (5000× *g* for 10 min), the residue was removed and the liquid phase was directly used for phytochemical investigation or taken to dryness in a rotary evaporator (bath temperature below 50 °C) and calculated the loss of drying. The extraction yield resulted 41.03, 28.92, 43.19 and 30.90 mg of dry extract from 1 g of fresh fruiting body, respectively, from substrates A–D. Extraction of samples deriving from each substrate (A–D) were performed in two independent extractions and the liquid phase from both extractions combined together and used as single sample.

As for the biological tests, an accurately weighted aliquot of extract was mixed in a defined volume of distilled water and solubilized in a sonicating bath at room temperature for one hour.

### 3.7. Untargeted LC-MS/MS-Based Metabolomics and Statistical Analysis

Untargeted metabolomics was carried out by using ultra-performance liquid chro- matography mass spectrometry (UHPLC)–QTOF employing a 1260 ultra-high-performance liquid chromatograph and a G6530A QTOF mass spectrometer (Agilent Technologies, Santa Clara, CA, USA). The chromatographic conditions are fully described in our recent paper [2].

Compound annotation was made using mummichog algorithm [51] implemented in “Functional analysis” module of MetaboAnalyst 5.0 [52] using 5 ppm of tolerance for both polarities. Heatmap, ANOVA and Functional Meta Analysis were also performed with MetaboAnalyst. For statistical analysis, samples were normalized by median, followed by pareto scaling.

### 3.8. Phenolic and Flavonoid Determination: HPLC-DAD-MS Analyses

The identification and quantification of selected phenolic compounds, namely gallic acid, benzoic acid, catechin, hydroxytyrosol, chlorogenic acid and epicatechin was carried out through HPLC-DA-MS analysis. The detailed protocol is included in a recent paper of ours [53].

### 3.9. Free Radical-Scavenging Activity

#### 3.9.1. DPPH Assay

The scavenging effect of mushroom extracts on DPPH radicals was evaluated spectrophotometrically according to literature [54,55,56].

#### 3.9.2. ABTS Assay

The ABTS radical cation scavenging activity was performed according to Re et al. [57] and Ozturk et al. [55].

#### 3.9.3. β-Carotene-Linoleic Acid Assay

The antioxidant activity of extracts was determined spectrophotometrically following the β-carotene–linoleic assay method of Yae et al. [58], Prieto et al. [59] and Vaz et al. [60].

### 3.10. Antimicrobial Tests

#### 3.10.1. Bacterial and Fungal Strains

The in vitro antimicrobial activity of extracts A–D samples was assessed against the following Gram-negative and Gram-positive bacterial strains: *Escherichia coli* (ATCC 10536), *E. coli* (PeruMycA 2), *E. coli* (PeruMycA 3), *Bacillus cereus* (PeruMycA 4), *Pseudomonas aeruginosa* (PeruMyc 5), *B. subtilis* (PeruMyc 6), *Salmonella typhy* (PeruMyc 7) and *Staphylococcus aureus* (ATCC 6538). Furthermore, the same extracts were assayed for the antifungal assays against different yeasts, dermatophyte and fungal pool species: *Candida albicans* (YEPGA 6183), *C. tropicalis* (YEPGA 6184), *C. albicans* (YEPGA 6379), *C. parapsilopsis* (YEPGA 6551), *Arthroderma crocatum* (CCF 5300), *A. curreyi* (CCF 5207), *A. gypseum* (CCF 6261), *A. insingulare* (CCF 5417), *A. quadrifidum* (CCF 5792), *Trichophyton mentagrophytes* (CCF 4823), *T. mentagrophytes* (CCF 5930), *T. rubrum* (CCF 4933), *T. rubrum* (CCF 4879) and *T. tonsurans* (CCF 4834), *Talaromyces* sp. (PMDB1), *Talaromyces minioluteus* (PMDB3), *Trichothecium roseum* (PMDB4), *Stemphylium vesicarium* (PMDB5), *Fusarium oxysporum* (PMDB6), *Auxarthron ostraviense* (PMDB7) and *Candida parapsilopsis* (PMDB10) [61].

#### 3.10.2. Antibacterial Activity

Determination of Minimum Inhibitory Concentration (MIC) was performed according to the broth dilution method M07-A9 drafted by the Clinical and Laboratory Standard Institute (CLSI M07-A9, 2012). The experimental conditions are reported in our previous paper [61].

#### 3.10.3. Antifungal Activity

Susceptibility testing against yeasts and filamentous fungi was performed according to the CLSI M27-A3 and M38-A2 protocols, respectively.

*Candida parapsilosis* (Ashford) Langeron & Talice (ATCC 22019) and *Candida krusei* (Castell.) Berkhout (ATCC 6258) strains were used as quality controls. The experimental conditions are reported in our previous paper [61].

For the mushroom extracts, the MIC end-points were defined as the lowest concentration that showed total growth inhibition.

#### 3.10.4. Statistical Analysis

The results were expressed as mean ± standard deviation and analyzed via Student test. The statistical significance was set a *p* < 0.05 and analysis was conducted through GraphPad Prism 5.01 version (GraphPad Software, San Diego, CA, USA).

## 4. Conclusions

The present findings support more in-depth investigations aimed at evaluating the influence of growth substrate on *P. columbinus* antimicrobial and antioxidant properties. The extracts from *P. columbinus* revealed valuable sources of primary and secondary metabolites, thus suggesting potential applications in the formulation of food supplements with biological properties, above all in terms of antioxidant and antimicrobial properties. Future studies are needed to further improve our knowledge of the metabolic pathways and the complexity of the compounds measured. This will also allow in understanding the mechanisms of action at the basis of the observed effects.

## Figures and Tables

**Table 1:** Quantitative analysis of phenolic and flavonoid compounds in *P. columbinus* extracts.

	Quantity (µg/mL ± S.D.)			
Extracts	A	B	C	D
Gallic acid	2.76 ± 0.12	1.29 ± 0.04	2.74 ± 0.05	1.97 ± 0.38
Hydroxytyrosol	not detected	not detected	4.43 ± 0.39	2.91 ± 0.77
Catechin	7.60 ± 0.21	12.25 ± 0.42	26.90 ± 1.07	14.54 ± 0.13
Chlorogenic acid	not detected	not detected	1.62 ± 0.13	1.10 ± 0.01
Epicatechin	not detected	5.69 ± 0.18	7.97 ± 0.19	11.98 ± 0.01
Benzoic acid	0.13 ± 0.02	0.06 ± 0.01	0.05 ± 0.01	0.015 ± 0.01

**Table 2:** Minimal inhibitory concentrations (MICs) of *P. columbinus* extracts against yeast, bacterial and dermatophytes strains.

	Extracts	MIC (µg mL^−1^) *				
		A	B	C	D	Fluconazole (µg/mL)
Yeasts	Strain (ID) *C. tropicalis* (YEPGA 6184)	125.99 (100–200)	79.37 (50–100)	200- > 200	200- > 200	2
	*C. albicans* (YEPGA 6379)	125.99 (100–200)	200- > 200	200- > 200	200- > 200	1
	*C. parapsilosis* (YEPGA 6551)	31.49 (25–50)	39.68 (25–59)	200- > 200	200- > 200	4
	*C. albicans* (YEPGA 6183)	62.99 (50–100)	158.74 (100–200)	200- > 200	200- > 200	2
	Gram -					Ciprofloxacin (µg/mL)
Bacteria	*E. coli* (ATCC 10536)	19.84 (12.5–25)	9.92 (6.25–12.5)	31.49 (25–50)	15.74 (12.5–25)	<0.12
	*E. coli* (PeryMycA 2)	158.74 (100–200)	79.27 (50–100)	9.92 (6.25–12.5)	19.84 (12.5–25)	1.23 (0.98–1.95)
	*E. coli* (PeruMycA 3)	200- > 200	200- > 200	79.37 (50–100)	125.99 (100–200)	0.62 (0.49–0.98)
	*P. aeruginosa* (PeruMycA 5)	62.99 (50–100)	62.99 (50–100)	125.99 (100–200)	125.99 (100–200)	1.23 (0.98–1.95)
	*S. typhy* (PeruMycA 7)	125.99 (100–200)	79.37 (50–100)	79.37 (50–100)	125.99 (100–200)	0.38 (0.24–0.49)
	*Gram +*					
	*B. cereus* (PeruMycA 4)	31.49 (25–50)	200- > 200	125.99 (100–200)	125.99 (100–200)	<0.12
	*B. subtilis* (PeruMycA 6)	79.37 (50–100)	79.37 (50–100)	125.99 (100–200)	158.74 (100–200)	<0.12
	*S. aureus* (ATCC 6538)	158.74 (100–200)	125.99 (100–200)	200- > 200	125.99 (100–200)	0.62 (0.98–0.49)
						Griseofulvin (µg/mL)
Dermatophytes	*T. mentagrophytes* (CCF 4823)	28.24 (12.5–25)	20.37 (6.25–12.5)	129.37 (50–100)	81.50 (25–50)	2.52 (2–4)
	*T. tonsurans* (CCF 4834)	129.37 (50–100)	112.99 (50–100)	89.68 (25–50)	112.99 (50–100)	0.198 (0.125–0.25)
	*T.rubrum* (CCF 4933)	158.74 (100–200)	200- > 200	16.17 (6.25–12.5)	158.74 (100–200)	1.26 (1–2)
	*A. quadrifidum* (CCF5792)	89.68 (25–50)	81.50 (25–50)	62.99 (50–100)	15.74 (12.5–25)	>8
	*T. erinacei* (CCF5930)	39.68 (25–50)	158.74 (100–200)	79.37 (50–100)	31.49 (25–50)	3.174 (2–4)
	*N. gypseum* (CCF6261)	31.49 (25–50)	125.99 (100–200)	79.37 (50–100)	62.99 (50–100)	1.587 (1–2)
	*A. currei* (CCF5207)	62.99 (50–100)	39.68 (25–50)	31.49 (25–50)	19.84 (12.5–25)	>8
	*A. insingulare* (CCF5417)	19.84 (12.5–25)	79.37 (50–100)	62.99 (50–100)	31.49 (25–50)	>8

* MIC values are reported as geometric means of three independent replicates (*n* = 3). MIC range concentrations are reported within brackets.

**Table 3:** Antioxidant properties of the tested extracts.

Extracts	DPPH Test EC50 (µg/mL ± SD)	ABTS Test EC50 (µg/mL ± SD)	Linoleic Assay EC50 (µg/mL ± SD)
A	4.98 ± 0.53 ^c^	6.16 ± 0.53 ^c^	11.29 ± 1.11 ^c^
B	2.25 ± 0.19 ^a^	4.34 ± 0.45 ^a^	8.47 ± 0.62 ^ab^
C	3.81 ± 0.32 ^c^	5.52 ± 0.51 ^c^	11.65 ± 0.99 ^c^
D	2.58 ± 0.21 ^ab^	4.61 ± 0.37 ^ab^	8.74 ± 0.86 ^ab^
Trolox (µg TE)	0.28 ± 0.03	0.66 ± 0.07	0.56 ± 0.06

Different letters indicated mean statistical differences (*p* < 0.05).

## Data Availability

The data presented in this study are available on request from the corresponding author.

## Supplementary Materials

The following are available online at https://www.mdpi.com/article/10.3390/antibiotics10101245/s1, Supplementary Tables S1: *P. columbinus* metabolites identified by untargeted HPLC-MS analysis; Tables S2: List of significance using post-hoc analysis (Fisher’s LSD); and Tables S3: Correlation matrix between phenolic and flavonoid compounds in *P. columbinus* extracts and antioxidant effects in selected experimental models.

## References

[1] Gargano M.L., van Griensven L.J., Isikhuemhen O.S., Lindequist U., Venturella G., Wasser S.P., Zervakis G.I. (2017). Medicinal mushrooms: Valuable biological resources of high exploitation potential. Plant Biosyst. Int. J. Deal. All Asp. Plant Biol..

[2] Ianni F., Blasi F., Angelini P., Di Simone S.C., Angeles Flores G., Cossignani L., Venanzoni R. (2021). Extraction Optimization by Experimental Design of Bioactives from Pleurotus ostreatus and Evaluation of Antioxidant and Antimicrobial Activities. Processes.

[3] Philippoussis A.N. (2009). Production of mushrooms using agro-industrial residues as substrates. Biotechnology for Agro-Industrial Residues Utilisation.

[4] Angelini P., Pagiotti R., Granetti B. (2008). Effect of antimicrobial activity of Melaleuca alternifolia essential oil on antagonistic potential of Pleurotus species against Trichoderma harzianum in dual culture. World J. Microbiol. Biotechnol..

[5] Lavelli V., Proserpio C., Gallotti F., Laureati M., Pagliarini E. (2018). Circular reuse of bio-resources: The role of Pleurotus spp. in the development of functional foods. Food Funct..

[6] Tsiantas K., Tsiaka T., Koutrotsios G., Siapi E., Zervakis G.I., Kalogeropoulos N., Zoumpoulakis P. (2021). On the identification and quantification of ergothioneine and lovastatin in various mushroom species: Assets and challenges of different analytical approaches. Molecules.

[7] Corrêa R.C.G., Brugnari T., Bracht A., Peralta R.M., Ferreira I.C. (2016). Biotechnological, nutritional and therapeutic uses of Pleurotus spp.(Oyster mushroom) related with its chemical composition: A review on the past decade findings. Trends Food Sci. Technol..

[8] Jayasuriya W., Handunnetti S.M., Wanigatunge C.A., Fernando G.H., Abeytunga D.T.U., Suresh T.S. (2020). Anti-inflammatory activity of Pleurotus ostreatus, a culinary medicinal mushroom, in wistar rats. Evid. Based Complementary Altern. Med..

[9] Jedinak A., Dudhgaonkar S., Wu Q.-l., Simon J., Sliva D. (2011). Anti-inflammatory activity of edible oyster mushroom is mediated through the inhibition of NF-κB and AP-1 signaling. Nutr. J..

[10] Khan N., Ajmal M., Nickten J., Aslam S., Ali M. (2013). Nutritional value of Pleurotus (Flabellatus) Djamor (R-22) cultivated on sawdusts of different woods. Pak. J. Bot..

[11] Llauradó G., Morris H.J., Lebeque Y., Gutiérrez A., Fontaine R., Bermúdez R.C., Perraud-Gaime I. (2013). Phytochemical screening and effects on cell-mediated immune response of Pleurotus fruiting bodies powder. Food Agric. Immunol..

[12] Maiti S., Mallick S.K., Bhutia S.K., Behera B., Mandal M., Maiti T.K. (2011). Antitumor effect of culinary-medicinal oyster mushroom, Pleurotus ostreatus (Jacq.: Fr.) P. Kumm., derived protein fraction on tumor-bearing mice models. Int. J. Med. Mushrooms.

[13] Sheng Y., Zhao C., Zheng S., Mei X., Huang K., Wang G., He X. (2019). Anti-obesity and hypolipidemic effect of water extract from Pleurotus citrinopileatus in C57 BL/6J mice. Food Sci. Nutr..

[14] Venturella G., Gargano M., Compagno R. (2015). The genus Pleurotus in Italy. Flora Mediterr..

[15] Wang H., Ng T. (2000). Isolation of a novel ubiquitin-like protein from Pleurotus ostreatus mushroom with anti-human immunodeficiency virus, translation-inhibitory, and ribonuclease activities. Biochem. Biophys. Res. Commun..

[16] Zhao S., Gao Q., Rong C., Wang S., Zhao Z., Liu Y., Xu J. (2020). Immunomodulatory effects of edible and medicinal mushrooms and their bioactive immunoregulatory products. J. Fungi.

[17] Patel Y., Naraian R., Singh V. (2012). Medicinal properties of Pleurotus species (oyster mushroom): A review. World J. Fungal Plant Biol..

[18] Adebayo E., Elkanah F., Afolabi F., Ogundun O., Alabi T., Oduoye O. (2021). Molecular characterization of most cultivated Pleurotus species in sub-western region Nigeria with development of cost effective cultivation protocol on palm oil waste. Heliyon.

[19] Bonatti M., Karnopp P., Soares H., Furlan S. (2004). Evaluation of Pleurotus ostreatus and Pleurotus sajor-caju nutritional characteristics when cultivated in different lignocellulosic wastes. Food Chem..

[20] Koutrotsios G., Kalogeropoulos N., Kaliora A.C., Zervakis G.I. (2018). Toward an increased functionality in oyster (Pleurotus) mushrooms produced on grape marc or olive mill wastes serving as sources of bioactive compounds. J. Agric. Food Chem..

[21] Yildiz S., Yildiz Ü.C., Gezer E.D., Temiz A. (2002). Some lignocellulosic wastes used as raw material in cultivation of the Pleurotus ostreatus culture mushroom. Process. Biochem..

[22] Owaid M.N., Abed I.A., Al-Saeedi S.S.S. (2017). Applicable properties of the bio-fertilizer spent mushroom substrate in organic systems as a byproduct from the cultivation of Pleurotus spp. Inf. Process. Agric..

[23] Kumla J., Suwannarach N., Sujarit K., Penkhrue W., Kakumyan P., Jatuwong K., Vadthanarat S., Lumyong S. (2020). Cultivation of mushrooms and their lignocellulolytic enzyme production through the utilization of agro-industrial waste. Molecules.

[24] Raman J., Jang K.Y., Oh Y.L., Oh M., Im J.H., Lakshmanan H., Sabaratnam V. (2020). Cultivation and Nutritional Value of Prominent *Pleurotus* spp.: An Overview. Mycobiology.

[25] Angelini P., Pagiotti R., Venanzoni R., Granetti B. (2009). Antifungal and allelopathic effects of Asafoetida against Trichoderma harzianum and Pleurotus spp. Allelopath. J..

[26] Carrasco-González J.A., Serna-Saldívar S.O., Gutiérrez-Uribe J.A. (2017). Nutritional composition and nutraceutical properties of the Pleurotus fruiting bodies: Potential use as food ingredient. J. Food Compos. Anal..

[27] Jeznabadi E.K., Jafarpour M., Eghbalsaied S. (2016). King oyster mushroom production using various sources of agricultural wastes in Iran. Int. J. Recycl. Org. Waste Agric..

[28] Kinge T., Adi E., Mih A., Ache N., Nji T. (2016). Effect of substrate on the growth, nutritional and bioactive components of Pleurotus ostreatus and Pleurotus florida. Afr. J. Biotechnol..

[29] Ritota M., Manzi P. (2019). Pleurotus spp. cultivation on different agri-food by-products: Example of biotechnological application. Sustainability.

[30] Guzmán G. (2000). Genus Pleurotus (Jacq.: Fr.) P. Kumm.(Agaricomycetideae): Diversity, taxonomic problems, and cultural and traditional medicinal uses. Int. J. Med. Mushrooms.

[31] Hiber O. (1982). Die Gattung Pleurotus (FR.) Kummer unter besonderer Berucksichtigung des Pleurotus eryngii-Formenkomplexes. Bibl. Mycol..

[32] Zervakis G., Balis C. (1996). A pluralistic approach in the study of Pleurotus species with emphasis on compatibility and physiology of the European morphotaxa. Mycol. Res..

[33] Sharma D., Singh V.P., Singh N.K. (2018). A Review on Phytochemistry and Pharmacology of Medicinal as well as Poisonous Mushrooms. Mini Rev. Med. Chem..

[34] Rodrigues Barbosa J., Dos Santos Freitas M.M., da Silva Martins L.H., de Carvalho Junior R.N. (2020). Polysaccharides of mushroom Pleurotus spp.: New extraction techniques, biological activities and development of new technologies. Carbohydr. Polym..

[35] Sharma A., Sharma A., Tripathi A. (2021). Biological activities of Pleurotus spp. polysaccharides: A review. J. Food Biochem..

[36] Elhusseiny S.M., El-Mahdy T.S., Awad M.F., Elleboudy N.S., Farag M.M.S., Aboshanab K.M., Yassien M.A. (2021). Antiviral, Cytotoxic, and Antioxidant Activities of Three Edible Agaricomycetes Mushrooms: Pleurotus columbinus, Pleurotus sajor-caju, and Agaricus bisporus. J. Fungi.

[37] Irshad A., Shahid M., Asghar M., Khan J.A. (2017). Antioxidant potential analysis of P. Ostreatus, P. Sajor-Caju, P. Sapidus and P. Columbinus. J. Biol. Regul. Homeost. Agents.

[38] Luo F., Zhong Z., Liu L., Igarashi Y., Xie D., Li N. (2017). Metabolomic differential analysis of interspecific interactions among white rot fungi Trametes versicolor, Dichomitus squalens and Pleurotus ostreatus. Sci. Rep..

[39] Bas C., Kuyper T.W., Noordeloos M., Vellinga E. (1990). Flora Agaricina Neerlandica—Critical Monographs on the Families of Agarics and Boleti Occurring in the Netherlands.

[40] Mišković J., Rašeta M., Čapelja E., Krsmanović N., Novaković A., Karaman M. (2021). Mushroom Species Stereum hirsutum as Natural Source of Phenolics and Fatty Acids as Antioxidants and Acetylcholinesterase Inhibitors. Chem. Biodivers..

[41] Soliman E.R., El-Sayed H. (2021). Molecular identification and antimicrobial activities of some wild Egyptian mushrooms: Bjerkandera adusta as a promising source of bioactive antimicrobial phenolic compounds. J. Genet. Eng. Biotechnol..

[42] Ferrante C., Recinella L., Ronci M., Menghini L., Brunetti L., Chiavaroli A., Leone S., Di Iorio L., Carradori S., Tirillini B. (2019). Multiple pharmacognostic characterization on hemp commercial cultivars: Focus on inflorescence water extract activity. Food Chem. Toxicol..

[43] Menghini L., Leporini L., Vecchiotti G., Locatelli M., Carradori S., Ferrante C., Zengin G., Recinella L., Chiavaroli A., Leone S. (2018). Crocus sativus L. stigmas and byproducts: Qualitative fingerprint, antioxidant potentials and enzyme inhibitory activities. Food Res. Int..

[44] Singer R. (1986). The Agaricales in Modern Taxonomy.

[45] Gams W., Hoekstra E., Aptroot A. (1998). CBS Course of Mycology.

[46] Stamets P. (2011). Growing Gourmet and Medicinal Mushrooms.

[47] Angelini P., Venanzoni R., Angeles Flores G., Tirillini B., Orlando G., Recinella L., Chiavaroli A., Brunetti L., Leone S., Di Simone S.C. (2020). Evaluation of antioxidant, antimicrobial and tyrosinase inhibitory activities of extracts from Tricholosporum goniospermum, an edible wild mushroom. Antibiotics.

[48] Edgar R.C. (2004). MUSCLE: A multiple sequence alignment method with reduced time and space complexity. BMC Bioinform..

[49] Kumar S., Stecher G., Li M., Knyaz C., Tamura K. (2018). MEGA X: Molecular evolutionary genetics analysis across computing platforms. Mol. Biol. Evol..

[50] Stecher G., Tamura K., Kumar S. (2020). Molecular evolutionary genetics analysis (MEGA) for macOS. Mol. Biol. Evol..

[51] Li S., Park Y., Duraisingham S., Strobel F.H., Khan N., Soltow Q.A., Jones D.P., Pulendran B. (2013). Predicting network activity from high throughput metabolomics. PLoS Comput. Biol..

[52] Pang Z., Chong J., Zhou G., de Lima Morais D.A., Chang L., Barrette M., Gauthier C., Jacques P.-É., Li S., Xia J. (2021). MetaboAnalyst 5.0: Narrowing the gap between raw spectra and functional insights. Nucleic Acids Res..

[53] di Giacomo V., Recinella L., Chiavaroli A., Orlando G., Cataldi A., Rapino M., Di Valerio V., Politi M., Antolini M.D., Acquaviva A. (2021). Metabolomic profile and antioxidant/anti-inflammatory effects of industrial hemp water extract in fibroblasts, keratinocytes and isolated mouse skin specimens. Antioxidants.

[54] Alexander B., Browse D., Reading S., Benjamin I. (1999). A simple and accurate mathematical method for calculation of the EC50. J. Pharmacol. Toxicol. Methods.

[55] Öztürk M., Duru M.E., Kivrak Ş., Mercan-Doğan N., Türkoglu A., Özler M.A. (2011). In vitro antioxidant, anticholinesterase and antimicrobial activity studies on three Agaricus species with fatty acid compositions and iron contents: A comparative study on the three most edible mushrooms. Food Chem. Toxicol..

[56] Shimada K., Fujikawa K., Yahara K., Nakamura T. (1992). Antioxidative properties of xanthan on the autoxidation of soybean oil in cyclodextrin emulsion. J. Agric. food Chem..

[57] Re R., Pellegrini N., Proteggente A., Pannala A., Yang M., Rice-Evans C. (1999). Antioxidant activity applying an improved ABTS radical cation decolorization assay. Free. Radic. Biol. Med..

[58] Shon M.-Y., Kim T.-H., Sung N.-J. (2003). Antioxidants and free radical scavenging activity of Phellinus baumii (Phellinus of Hymenochaetaceae) extracts. Food Chem..

[59] Prieto M., Rodríguez-Amado I., Vázquez J.A., Murado M. (2012). β-Carotene assay revisited. Application to characterize and quantify antioxidant and prooxidant activities in a microplate. J. Agric. Food Chem..

[60] Vaz J.A., Barros L., Martins A., Santos-Buelga C., Vasconcelos M.H., Ferreira I.C. (2011). Chemical composition of wild edible mushrooms and antioxidant properties of their water soluble polysaccharidic and ethanolic fractions. Food Chem..

[61] Angelini P., Matei F., Flores G.A., Pellegrino R.M., Vuguziga L., Venanzoni R., Tirillini B., Emiliani C., Orlando G., Menghini L. (2021). Metabolomic Profiling, Antioxidant and Antimicrobial Activity of Bidens pilosa. Processes.

