# Supplementary material for: Metabolomic Profiling and Biological Activities of Pleurotus columbinus Quél. Cultivated on Different Agri-Food Byproducts
Source: Antibiotics (Basel). 2021 Oct 14;10(10):1245. doi: 10.3390/antibiotics10101245 (PMC8532797; doi:10.3390/antibiotics10101245)
Supplement: Supplementary file 1 [file antibiotics-10-01245-s001.zip › antibiotics-1394191-supplementary.pdf]

Table S1. *P. columbinus* metabolites identified by untargeted HPLC-MS analysis.

Metabolite name	KEGG	HMDB	PubChem	ChEBI	METLIN
**amino acids pathway**					
L-Alanine	C00041	HMDB0000161	5950	16977	NA
L-Lysine	C00047	HMDB0000182	5962	18019	5200
L-Arginine	C00062	HMDB0000517	6322	16467	5502
L-Glutamine	C00064	HMDB0000641	5961	18050	5614
L-Serine	C00065	HMDB0000187	5951	17115	5203
L-Methionine	C00073	HMDB0000696	6137	16643	5664
Ornithine	C00077	HMDB0000214	6262	15729	27
L-Phenylalanine	C00079	HMDB0000159	6140	17295	28
L-Tyrosine	C00082	HMDB0000158	6057	17895	34
L-Leucine	C00123	HMDB0000687	6106	15603	24
L-Histidine	C00135	HMDB0000177	6274	15971	21
L-Proline	C00148	HMDB0000162	145742	17203	29
L-Asparagine	C00152	HMDB0000168	6267	17196	14
L-Valine	C00183	HMDB0000883	6287	16414	5842
L-Threonine	C00188	HMDB0000167	6288	16857	32
L-Glutamic acid	C00025	HMDB0000148	33032	16015	5174
L-Aspartic acid	C00049	HMDB0000191	5960	17053	5206
L-Homocysteine	C00155	HMDB0000742	91552	17588	3256
Citrulline	C00327	HMDB0000904	9750	16349	16
L-Cystine	C00491	HMDB0000192	67678	16283	5207
**Modified aminoacids**					
5-Hydroxy-L-tryptophan	C00643	HMDB0000472	439280	17780	NA
L-Histidinol phosphate	C01100	METPA0128	NA	16996	NA
L-Glutamic acid 5-phosphate	C03287	HMDB0001228	193475	17798	6093
Methionine sulfoxide	C02989	HMDB0002005	158980	17016	6428
N-Acetylglutamic acid	C00624	HMDB0001138	70914	17533	6031
4-Acetamidobutanoic acid	C02946	HMDB0003681	18189	17645	NA
Argininosuccinic acid	C03406	HMDB0000052	16950	15682	5115
gamma-Glutamyl-beta-ami-nopropiononitrile	C06114	HMDB0060477	440920	28092	NA
**Fatty acids pathway**					
Palmitic acid	C00249	HMDB0000220	985	15756	187
Stearic acid	C01530	HMDB0000827	5281	28842	189
Oleic acid	C00712	HMDB0000207	445639	16196	190
Gamma-Linolenic acid	C06426	HMDB0003073	5280933	28661	386
Docosahexaenoic acid	C06429	HMDB0002183	445580	28125	3457
8,11,14-Eicosatrienoic acid	C03242	HMDB0002925	5280581	53486	259
**Vitamins pathway**					
Riboflavin	C00255	HMDB0000244	493570	17015	5249
Biotin	C00120	HMDB0000030	171548	15956	243
Thiamine	C00378	HMDB0000235	1130	18385	5242
Thiamine monophosphate	C01081	HMDB0002666	3382778	37574	3488
Thiamine pyrophosphate	C00068	HMDB0001372	1132	9532	2832
Folic acid	C00504	HMDB0000121	6037	27470	246
5,10-Methylene-THF	C00143	HMDB0001533	439175	15636	6304
N10-Formyl-THF	C00234	HMDB0000972	1,4E+08	15637	5912
Tetrahydrofolic acid	C00101	HMDB0001846	91443	20506	714

**5-Methyltetrahydrofolate pathway**	C00440	HMDB0001396	439234	15641	6215
THF-L-glutamate	C09332	HMDB0006825	442163	28624	NA
Pantothenic acid	C00864	HMDB0000210	6613	46905	NA
Nicotinic acid mononucleotide	C01185	HMDB0001132	5,3E+07	15763	6026
Nicotinamide riboside	C03150	HMDB0000855	439924	15927	5818
Pyridoxine	C00314	HMDB0000239	1054	16709	5245
Pyridoxamine 5'-phosphate	C00647	HMDB0001555	1053	18335	NA
**Modified vitamins**					
Dethiobiotin	C01909	HMDB0003581	445027	42280	3351
**Monosaccharides pathway**					
D-Glucose	C00031	NA	NA	NA	NA
D-Ribose	C00121	HMDB0000283	5779	47013	313
**Oligosaccharides pathway**					
Raffinose	C00492	HMDB0003213	439242	16634	138
Stachyose	C01613	HMDB0003553	439531	17164	6951
Sucrose	C00089	HMDB0000258	5988	17992	137
Trehalose 6-phosphate	C00689	HMDB0001124	122336	18283	6019
**Sugar acids pathway**					
Glucuronic acid	C00257	HMDB0000625	10690	33198	345
**Modified polysaccharides pathway**					
(GlcNAc)2 (Man)2 (PP-Dol)1	(GlcNAc)2 (Man)2 (PP-Dol)1	(GlcNAc)2 (Man)2 (PP-Dol)1	(GlcNAc)2 (Man)2 (PP-Dol)1	(GlcNAc)2 (Man)2 (PP-Dol)1	(GlcNAc)2 (Man)2 (PP-Dol)1
(GlcNAc)2 (Man)3 (PP-Dol)1	(GlcNAc)2 (Man)3 (PP-Dol)1	(GlcNAc)2 (Man)3 (PP-Dol)1	(GlcNAc)2 (Man)3 (PP-Dol)1	(GlcNAc)2 (Man)3 (PP-Dol)1	(GlcNAc)2 (Man)3 (PP-Dol)1
(GlcN)1 (Ino(acyl)-P)1 (Man)1	(GlcN)1 (Ino(acyl)-P)1 (Man)1	(GlcN)1 (Ino(acyl)-P)1 (Man)1	(GlcN)1 (Ino(acyl)-P)1 (Man)1	(GlcN)1 (Ino(acyl)-P)1 (Man)1	(GlcN)1 (Ino(acyl)-P)1 (Man)1
(GlcN)1 (Ino(acyl)-P)1 (Man)2	(GlcN)1 (Ino(acyl)-P)1 (Man)2	(GlcN)1 (Ino(acyl)-P)1 (Man)2	(GlcN)1 (Ino(acyl)-P)1 (Man)2	(GlcN)1 (Ino(acyl)-P)1 (Man)2	(GlcN)1 (Ino(acyl)-P)1 (Man)2
**Shikimate pathway**					
Shikimic acid	C00493	HMDB0003070	8742	16119	338
Shikimate 3-phosphate	C03175	METPA0369	NA	17052	NA
3-Dehydroquinate	C00944	HMDB0012710	439351	17947	NA
4-Amino-4-deoxychorismate	C11355	METPA0993	NA	18198	NA
**Mevalonate pathway**					
Acetoacetyl-CoA	C00332	HMDB0001484	439214	15345	449
Geranylgeranyl-PP	C00353	HMDB0004486	447277	48861	NA
Mevalonic acid-5P	C01107	HMDB0001343	439400	17436	6177
Farnesol	C06081	HMDB0004305	445070	16619	7048
(+)-7-Isojasmonic acid CoA	C16339	HMDB0060298	7,1E+07	NA	NA
All-trans-hexaprenyl diphosphate	C01230	HMDB0012188	5280413	17528	NA
**Glycolytic pathway**					
Pyruvic acid	C00022	HMDB0000243	1060	32816	117
Fructose 6-phosphate	C00085	HMDB0000124	69507	15946	5159
Dihydroxyacetone phosphate	C00111	HMDB0001473	668	16108	6262
Glyceric acid 1,3-biphosphate	C00236	HMDB0001270	439191	16001	NA
Dolichol b-D-glucosyl phosphate	C01246	HMDB0001054	2,3E+07	15812	5969
Uridine diphosphate glucose	C00029	HMDB0000286	8629	46229	5278

3-Phosphoglycerate	C00197	NA	NA	NA	NA
**Pentose phosphate pathway**					
Ribose 5-phosphate	C00117	NA	NA	NA	NA
Phosphoribosyl pyrophosphate	C00119	HMDB0000280	7339	17111	5274
Deoxyribose 5-phosphate	C00673	HMDB0001031	4,6E+07	16132	5956
**Calvin cycle**					
3-Phosphoglycerate	C00197	NA	NA	NA	NA
**Mannose pathway**					
Dolichol phosphate D-mannose	G10617	NA	NA	NA	NA
Guanosine diphosphate mannose	C00096	HMDB0001163	18396	15820	6045
**Biosynthesis of Triacylglycerols**					
Glycerol 3-phosphate	C00093	HMDB0000126	439162	15978	5161
**Tricarboxylic acid cycle**					
Citric acid	C00158	HMDB0000094	311	30769	124
**Glyoxylate cycle**					
cis-Aconitic acid	C00417	HMDB0000072	643757	32805	5130
**Purine methabolism**					
Hypoxanthine	C00262	HMDB0000157	790	17368	83
Xanthylic acid	C00655	HMDB0001554	73323	15652	NA
SAICAR	C04823	HMDB0000797	160666	18319	5762
**Nucleotide biosynthesis**					
Guanine	C00242	HMDB0000132	764	16235	315
Cytosine	C00380	HMDB0000630	597	16040	283
5-Methylcytosine	C02376	HMDB0002894	65040	27551	3247
Uracil	C00106	HMDB0000300	1174	17568	258
Adenosine	C00212	HMDB0000050	60961	16335	86
Deoxyadenosine monophosphate	C00360	HMDB0000905	12599	17713	3461
ADP	C00008	HMDB0001341	6022	16761	6175
dADP	C00206	HMDB0001508	5,3E+07	16174	6286
Deoxyadenosine	C00559	HMDB0000101	13730	17256	3382
Uridine	C00299	HMDB0000296	6029	16704	90
Uridine 5'-diphosphate	C00015	HMDB0000295	6031	17659	NA
Uridine diphosphate-N-acetylglu-cosamine	C00043	HMDB0000290	445675	16264	5281
Uridine 5'-monophosphate	C00105	HMDB0000288	6030	16695	NA
Deoxyuridine	C00526	HMDB0000012	13712	16450	5086
Cytidine	C00475	HMDB0000089	6253	17562	3376
Cytidine monophosphate	C00055	HMDB0000095	6131	17361	5143
Guanosine monophosphate	C00144	HMDB0001397	6804	17345	6216
Adenosine phosphosulfate	C00224	HMDB0001003	10238	17709	5933
dCMP	C00239	HMDB0001202	13945	15918	6078
Deoxyadenosine monophosphate	C00360	HMDB0000905	12599	17713	3461
dTDP	C00363	HMDB0001274	164628	18075	6129
Cyclic GMP	C00942	HMDB0001314	24316	16356	6152
Diadenosine tetraphosphate	C01260	HMDB0001211	21706	17422	NA
Inosine	C00294	HMDB0000195	6021	17596	84
Deoxyuridine triphosphate	C00460	HMDB0001191	65070	17625	6069
**hydroxylation of phenylalanine to tyrosine**					
4a-Carbinolamine tetrahydrobiop-terin	C00268	HMDB0002215	1,4E+08	43120	6552
Tetrahydrobiopterin	C00272	HMDB0000027	44257	59560	5098

**pathway of structural phospho-lipids**					
Citicoline	C00307	HMDB0001413	13804	16436	6229
**Aminoacids pathway**					
N-Acetylornithine	C00437	HMDB0003357	439232	16543	NA
O-Phosphohomoserine	C01102	HMDB0003484	151187	15961	NA
N-Acetyl-L-glutamate 5-semialde-hyde	C01250	HMDB0006488	192878	16319	NA
4-Hydroxy-L-threonine	C06056	METPA0747	NA	28330	NA
**Lysine degradation**					
Oxoadipic acid	C00322	HMDB0000225	71	15753	5234
Saccharopine	C00449	HMDB0000279	160556	16927	383
Aminoadipic acid	C00956	HMDB0000510	469	37024	5496
**L-leucine biosynthesis**					
Isopropylmaleate	C02631	HMDB0012241	5280533	17275	NA
L-2-Aminoadipate adenylate	C05560	HMDB0006941	5,3E+07	NA	NA
**GABA shunt**					
Gamma-Aminobutyric acid	C00334	HMDB0000112	223130	16865	NA
**Biochemical synthesis of glyco-sylated proteins and lipids – Chi-tin synthesis**					
Glucosamine	C00329	HMDB0001514	439213	47977	266
Glucosamine 6-phosphate	C00352	HMDB0001254	439217	15873	6111
N-Acetyl-D-Glucosamine 6-Phos-phate	C00357	HMDB0001062	440996	15784	5975
Chitin	C00461	HMDB0003362	444514	71404	6903
**UMP(uridine-5′-monofosfate) bi-osynthesis**					
4,5-Dihydroorotic acid	C00337	HMDB0000528	648	30865	5513
**Metylxanthine biosynthesis (caf-feine/theobromine)**					
Xanthine	C00385	HMDB0000292	1188	17712	82
**Porphyrin pathway**					
5-Aminolevulinic acid	C00430	HMDB0001149	137	17549	6037
Uroporphyrinogen III	C01051	HMDB0001086	1179	15437	79
Protoporphyrinogen IX	C01079	HMDB0001097	121893	15435	6003
Porphobilinogen	C00931	HMDB0000245	1021	17381	76
Protoporphyrin IX	C02191	HMDB0000241	NA	15430	4158
Siroheme	C00748	METPA0076	NA	28599	NA
Coproporphyrin III	C05770	HMDB0000570	NA	27609	5553
Precorrin 2	C02463	METPA0294	NA	50602	NA
**NAD biosynthesis**					
Nicotinamide ribotide	C00455	HMDB0000229	14180	16171	5238
**Transaminations**					
Pyridoxamine	C00534	HMDB0001431	1052	16410	238
**Multi Pathways**					
S-Adenosylhomocysteine	C00021	HMDB0000939	439155	16680	296
Oxoglutaric acid	C00026	HMDB0000208	51	30915	5218
3-methyl pyruvic acid	C00109	HMDB0000005	58	30831	NA
Alpha-ketoisovaleric acid	C00141	HMDB0000019	49	16530	5091
N-Acetyl-D-glucosamine	C00140	HMDB0000215	439174	506227	3356
4-Trimethylammoniobutanal	C01149	HMDB0001345	133	18020	6179
Lauroyl-CoA	C01832	HMDB0003571	165436	15521	6959

Long-chain acyl-CoA	C02843	MA3TEM004	5783	33184	NA
**Sterol pathway**					
Cholesterol	C00187	HMDB0000067	5997	1307929	163
Fecosterol	C04525	METPA0507	NA	17038	NA
**Aminobenzoic acid**					
2-Aminobenzoic acid	C00108	HMDB0001123	227	30754	6018
**Sugar alcohol pathway**					
Glycerol	C00116	HMDB0000131	753	17754	105
Xylitol	C00379	HMDB0002917	6912	17151	NA
Mannitol	C00392	HMDB0000765	6251	16899	142
**Polyamine pathway**					
Putrescine	C00134	HMDB0001414	1045	17148	3226
Aminopropylcadaverine	C16565	HMDB0012189	65523	64860	NA
Spermidine	C00315	HMDB0001257	1102	16610	254
**Polyamine biosynthesis**					
S-Adenosylmethioninamine	C01137	HMDB0000988	439415	15625	3501
**Coenzyme**					
Oxidized glutathione	C00127	HMDB0003337	65359	17858	6893
Niacinamide	C00153	HMDB0001406	936	17154	1497
**Peptides of nonprotein origin**					
Carnosine	C00386	HMDB0000033	439224	15727	38
**Oligopeptides pathway**					
Ophthalmic acid					
**Putrescine, spermine and spermi-dine degradation**					
4-Aminobutyraldehyde	C00555		NA	NA	NA
**phospholipid biosynthesis**					
Phosphorylcholine	C00588	HMDB0001565	1014	18132	6326
**L-tryptophan methabolism**					
Indoleacetaldehyde	C00637	HMDB0001190	800	18086	6068
Cinnavalininate	C05640	HMDB0004078	114918	NA	NA
5-Hydroxy-N-formylkynurenine	C05648	HMDB0004086	440744	2065	NA
**sphingolipid biosynthesis**					
Sphinganine	C00836	HMDB0000269	91486	16566	5268
**pyrimidine deoxyribonucleosides salvage**					
Deoxycytidine	C00881	HMDB0000014	13711	15698	3367
**synthesis of leukotriene**					
Leukotriene A4	C00909	HMDB0001337	5280383	15651	3449
Leukotriene B4	C02165	HMDB0001085	5283128	15647	406
**dihydrofolate synthesis**					
7,8-Dihydropteroic acid	C00921	HMDB0001412	170	4581	6228
**formaldehyde oxidation**					
S-Formylglutathione	C01031	HMDB0001550	189122	16225	3469
**gamma amino acids pathway**					
4-Guanidinobutanoic acid	C01035	HMDB0003464	500	15728	6938
**prosthetic group of several acyl carrier proteins**					
Pantetheine 4'-phosphate	C01134	HMDB0001416	987	16858	NA
**glycerolipids pathway**					
Glycerylphosphorylethanolamine	C01233	HMDB0000114	123874	52330	5151
**aminoadipic pathway**					
Homocitric acid	C01251	HMDB0003518	439459	52222	6943

**dipeptide**					
Anserine	C01262	HMDB0000194	112072	18323	5209
**lipid-dependent phytate biosynthesis**					
Inositol 1,3,4,5,6-pentakisphosphate	C01284	HMDB0003529	NA	16322	NA
**sphingolipid biosynthesis**					
Ergosterol	C01694	HMDB0000878	2,2E+07	16933	5839
**γ-glutamyl cycle**					
Pyroglutamic acid	C01879	HMDB0000267	7405	18183	3251
**thiol metabolism**					
Trypanothione	C02090	HMDB0060520	449517	17842	NA
**homocysteine and cysteine interconversion**					
L-Cystathionine	C02291	HMDB0000099	439258	17482	39
**hydroxy fatty acids pathway**					
2-Isopropylmalic acid	C02504	HMDB0000402	5280523	35128	339
**pyridine nucleotide cycling**					
Nicotinate D-ribonucleoside	C05841	HMDB0006809	161234	27748	NA
**L- arginine degradation**					
4-Guanidinobutanamide	C03078	METPA0359	NA	18062	NA
**5-aminoimidazole ribonucleotide biosynthesis**					
5-Phosphoribosylamine	C03090	HMDB0001128	439905	37737	6023
**cyclic peptides**					
Trypanothione disulfide	C03170	HMDB0060521	115098	35490	NA
**L-tryptophan degradation**					
	C03227	HMDB0011631	11811	17380	NA
**L-isoleucine degradation**					
2-Methylacetoacetyl-CoA	C03344	HMDB0001157	53	15476	6040
**biosynthesis of purine nucleotides and thiamin.**					
5-Aminoimidazole ribonucleotide	C03373	HMDB0001235	161500	138560	6097
**glutathione-mediated detoxification**					
S-Lactoylglutathione	C03451	HMDB0001066	440018	15694	5979
**coenzyme A biosynthesis**					
D-4'-Phosphopantothenate	C03492	HMDB0001016	41635	15905	3427
**tricarboxylic acids and derivatives**					
but-1-ene-1,2,4-tricarboxylic acid	C04002	HMDB0060320	5280640	17516	NA
**carboxamide pathway**					
5-Aminoimidazole-4-carboxamide	C04051	HMDB0003192	9679	2030	629
**L-arginine/L-ornithine biosynthesis**					
N-Acetyl-L-glutamyl 5-phosphate	C04133	HMDB0006456	440236	16878	3396
**methionine biosynthesis**					
5-Methylthioribose 1-phosphate	C04188	HMDB0000963	5,3E+07	27859	5909
**flavin biosynthesis**					
6,7-Dimethyl-8-(1-D-ribityl)lumazine	C04332	HMDB0003826	168989	17601	NA
5-Amino-6-ribitylamino uracil	C04732	HMDB0011106	193516	15934	NA
**purine metabolism / conversion of glutamine to glutamate**					

N(2)-Formyl-N(1)-(5-phospho-D-ribosyl)glycinamide	C04376	HMDB0001308	1,6E+07	NA	NA
**pyrimidine nucleosides pathway**					
5,10-Methylenetetrahydrometh-anopterin	C04377	HMDB0060401	440314	16568	NA
**alpha amino acids pathway**					
2-Amino-3-carboxymuconic acid semialdehyde	C04409	HMDB0001330	5280673	995	6165
**riboflavin metabolism**					
5-Amino-6-(5'-phosphoribityla-mino)uracil	C04454	HMDB0003841	1,9E+07	18247	NA
**dehydroquinate biosynthesis**					
2-Dehydro-3-deoxy-D-arabino-heptonate 7-phosphate	C04691	METPA0533	NA	18150	NA
**inosine-5'-phosphate biosynthesis**					
Phosphoribosyl formamidocarbox-amide	C04734	HMDB0001439	166760	18381	6247
**hydroxyeicosatetraenoic acid**					
15(S)-HETE	C04742	HMDB0003876	9966861	15558	NA
**purine metabolism**					
5-amino-1-(5-phospho-D-ribo-syl)imidazole-4-carboxylate	C04751	HMDB0006273	165388	28413	NA
**tetrahydrofolate biosynthesis**					
7,8-Dihydroneopterin	C04874	HMDB0002275	65074	17001	6588
**sterol biosynthesis**					
4,4-Dimethyl-5a-cholesta-8,24-dien-3-b-ol	C05108	HMDB0001286	5,1E+07	18364	6135
**histidine metabolism**					
Imidazole-4-acetaldehyde	C05130	HMDB0003905	150841	27398	NA
**lipid metabolism**					
(2E)-Decenoyl-CoA	C05275	HMDB0003948	5280768	10723	NA
**Calvin-Benson-Bassham cycle / pentose phosphate pathway**					
D-Sedoheptulose 7-phosphate	C05382	HMDB0001068	9,2E+07	133983	5980
**nucleoside pathway**					
Deoxyinosine	C05512	HMDB0000071	65058	28997	3383
**catechols pathway**					
3,4-Dihydroxyphenylglycol	C05576	HMDB0000318	91528	1387	5307
3,4-Dihydroxymandelic acid	C05580	HMDB0001866	85782	27637	696
**phenylalanine metabolism**					
N-Acetyl-D-phenylalanine	C05620	METPA0627	NA	28203	NA
**indole-3-acetic acid derivative pathway**					
5-Hydroxyindoleacetic acid	C05635	HMDB0000763	1826	27823	2975
**beta-alanine biosynthesis**					
3-Aminopropionaldehyde	C05665	HMDB0001106	75	18090	6007
**Eme biosynthesis**					
Sirohydrochlorin	C05778	METPA0681	NA	18023	NA
Heme	C00032	HMDB0003178	26945	17627	3680
Heme O	C15672	HMDB0001162	0	24480	6044
**histamine pathway**					
Methylimidazoleacetic acid	C05828	HMDB0002820	75810	1606	3774

**polyprenyl diphosphates pathway**					
Dehydrodolichol diphosphate	C05859	NA	NA	NA	NA
**pentose phosphates pathway**					
2,5-Diaminopyrimidine nucleoside triphosphate	C05923	HMDB0006821	440841	929	NA
**metabolism of amino groups**					
N4-Acetylaminobutanal	C05936	HMDB0004226	440850	7386	NA
**sphingolipid biosynthesis**					
Phytosphingosine	C12144	HMDB0004610	122121	46961	7066
**glutathione-mediated detoxification**					
S-(Hydroxymethyl)glutathione	C14180	HMDB0004662	447123	48926	7068
**serotonin and melatonin biosynthesis**					
4a-Hydroxytetrahydrobiopterin	C15522	HMDB0002281	1,4E+08	15642	6591
**sterol biosynthesis**					
Chenodeoxyglycocholoyl-CoA	C15670	HMDB0006897	440685	NA	NA
**3-oxo-acyl coas pathway**					
3-Oxo-OPC6-CoA	C16334	HMDB0060376	7,1E+07	NA	NA
**dipeptides pathway**					
gamma-L-Glutamyl-L-2-aminobutyrate	C21015	NA	2,5E+08	NA	NA
**Miscellaneous**					
2,5-Diamino-6-(5-phospho-D-ribitylamino)pyrimidin-4(3H)-one	C18910	NA	1,2E+08	52402	NA
Sulfate	C00059	HMDB0001448	1117	26836	3233
Ethanolamine	C00189	HMDB0000149	700	16000	3207
Glyceric acid	C00258	HMDB0000139	439194	32398	280
Phenylacetaldehyde	C00601	HMDB0006236	998	16424	NA
Glycerophosphocholine	C00670	HMDB0000086	71920	16870	370
Diacetyl	C00741	HMDB0003407	650	16583	6921
Serotonin	C00780	HMDB0000259	5202	28790	74
4-Amino-5-hydroxymethyl-2-methylpyrimidine	C01279	METPA0166	NA	16892	NA
2-Amino-4-hydroxy-6-hydroxymethyl-7,8-dihydropteridine	C01300	METPA0169	NA	17083	NA
2,5-Diamino-6-(5'-phosphoribosylamino)-4-pyrimidineone	C01304	METPA0171	NA	29114	NA
Melatonin	C01598	HMDB0001389	896	16796	73
Deoxyribose	C01801	HMDB0003224	2,3E+07	28816	3258
2-Oxosuccinamic acid	C02362	HMDB0060350	439716	16327	NA
2-Phenylacetamide	C02505	HMDB0010715	7680	16562	NA
Indoleglycerol phosphate	C03506	METPA0401	NA	18299	NA
PhosphoribosylformiminoAICAR-phosphate	C04896	HMDB0012277	440534	18302	NA
Homoisocitrate	C05662	METPA0637	NA	15404	NA
2S-acetyl-2-hydroxy-butanoic acid	C06006	HMDB0006900	440875	27681	NA
D-erythro-3-Methylmalate	C06032	METPA0739	NA	27394	NA
2-Oxo-3-hydroxy-4-phosphobutanoic acid	C06054	HMDB0006801	2,1E+07	27951	NA

Table S2. List of significance using post-hoc analysis (Fisher's LSD).

Metabolite name	f.value	p.value	-log10(p)	FDR	Fisher's LSD
3-Dehydroquinate	574.38	6.9139E-15	14.16	1.6109E-12	B - A; C - A; B - C; B - D; C - D
5,10-Methylenetetrahydromethanopterin	405.37	7.7332E-14	13.112	9.0091E-12	D - A; D - B; D - C
gamma-L-Glutamyl-L-2-aminobutyrate	244.85	2.4947E-12	11.603	1.9375E-10	B - A; C - A; D - A; B - C; B - D
Siroheme	196.17	1.1374E-11	10.944	5.6695E-10	C - A; D - A; C - B; D - B; D - C
Biotin	194.24	1.2166E-11	10.915	5.6695E-10	A - B; C - A; A - D; C - B; C - D
Protoporphyrin IX	180.89	1.9772E-11	10.704	7.678E-10	B - A; C - A; C - B; B - D; C - D
4-Guanidinobutanamide	146.6	8.2418E-11	10.084	2.7434E-09	A - B; A - C; D - A; D - B; D - C
(2E)-Decenoyl-CoA	141.97	1.0241E-10	9.9896	2.8881E-09	D - A; D - B; D - C
S-Adenosylhomocysteine	139.77	1.1383E-10	9.9437	2.8881E-09	A - B; A - C; A - D
Pantothenic acid	138.02	1.2395E-10	9.9067	2.8881E-09	B - A; C - A; B - D; C - D
Trehalose 6-phosphate	112.65	4.8657E-10	9.3129	1.0306E-08	D - A; D - B; D - C
Cytidine monophosphate	109.62	5.8396E-10	9.2336	1.1339E-08	D - A; D - B; D - C
Pyridoxamine 5'-phosphate	107.16	6.7973E-10	9.1677	1.2183E-08	B - A; C - A; D - A
15(S)-HETE	105.54	7.5276E-10	9.1233	1.2528E-08	B - A; C - A; D - A; B - C; D - C
ADP	94.704	1.55E-09	8.8097	2.4077E-08	C - A; C - B; C - D
L-Glutamine	93.289	1.7132E-09	8.7662	2.4949E-08	C - A; A - D; C - B; B - D; C - D
N-Acetylglutamic acid	87.072	2.7071E-09	8.5675	3.7103E-08	A - B; A - D; B - D; C - D
L-Valine	86.159	2.9028E-09	8.5372	3.7575E-08	D - A; D - B; D - C
Cholesterol	80.823	4.4283E-09	8.3538	5.4305E-08	D - A; D - B; D - C
Sucrose	77.576	5.8011E-09	8.2365	6.6383E-08	D - A; D - B; D - C
5-Phosphoribosylamine	77.213	5.983E-09	8.2231	6.6383E-08	D - A; D - B; D - C
Stachyose	71.691	9.7352E-09	8.0117	1.0311E-07	D - A; D - B; D - C
3-methyl pyruvic acid	67.678	1.4184E-08	7.8482	1.4369E-07	A - B; A - D; C - B; B - D; C - D
Porphobilinogen	62.856	2.2938E-08	7.6394	2.2269E-07	B - A; C - A; D - A
4-Amino-5-hydroxymethyl-2-methylpyrimidine	61.413	2.6664E-08	7.5741	2.4851E-07	D - A; D - B; D - C
4-Trimethylammoniobutanal	59.031	3.4435E-08	7.463	2.994E-07	A - B; A - C; A - D
Thiamine pyrophosphate	58.963	3.4695E-08	7.4597	2.994E-07	D - A; D - B; D - C
SAICAR	57.683	3.9967E-08	7.3983	3.3258E-07	B - A; D - A; B - C; D - B; D - C
Phosphoribosyl formamidocarboxamide	53.313	6.629E-08	7.1785	5.3261E-07	B - A; C - A; B - D; C - D
but-1-ene-1,2,4-tricarboxylic acid	52.289	7.5051E-08	7.1246	5.829E-07	B - A; C - A; D - A
Gluconic acid	51.425	8.3473E-08	7.0785	6.2739E-07	A - B; A - C; A - D
4-Hydroxy-L-threonine	47.79	1.3306E-07	6.876	9.6054E-07	A - B; A - C; A - D
Farnesol	47.623	1.3604E-07	6.8663	9.6054E-07	A - B; A - C; A - D
Pantetheine 4'-phosphate	43.012	2.5855E-07	6.5874	1.7719E-06	B - A; B - C; B - D
Spermidine	40.182	3.9563E-07	6.4027	2.5988E-06	B - A; C - A; B - D; C - D
2-Aminobenzoic acid	40.015	4.0602E-07	6.3915	2.5988E-06	A - B; A - D; C - B

2-Amino-4-hydroxy-6-hydroxymethyl-7,8-dihydropteridine	39.91	4.1269E-07	6.3844	2.5988E-06	C - A; A - D; C - B; B - D; C - D
Deoxyinosine	37.226	6.354E-07	6.197	3.896E-06	A - D; B - D; C - D
Heme O	35.402	8.6557E-07	6.0627	5.075E-06	B - A; D - A; B - C; D - C
(GlcNAc)2 (Man)3 (PP-Dol)1	35.364	8.7124E-07	6.0599	5.075E-06	B - A; D - A; B - C; D - C
Citicoline	35.033	9.2299E-07	6.0348	5.2453E-06	A - B; A - D; C - B; C - D
5-Hydroxy-N-formylkynurenine	31.221	1.8597E-06	5.7306	1.0317E-05	A - B; A - D; C - B; C - D
L-Histidinol phosphate	31.058	1.9191E-06	5.7169	1.0399E-05	A - D; B - D; C - D
THF-L-glutamate	30.928	1.9683E-06	5.7059	1.0423E-05	A - B; D - A; D - B; D - C
5-amino-1-(5-phospho-D-ribosyl)imidazole-4-carboxylate	29.478	2.6262E-06	5.5807	1.3598E-05	A - B; A - C; A - D
Oxoadipic acid	28.925	2.9411E-06	5.5315	1.4897E-05	A - B; A - D; C - B; C - D
5-Methylthioribose 1-phosphate	28.738	3.0572E-06	5.5147	1.5156E-05	B - A; D - A; D - C
Glycerol	27.509	3.9639E-06	5.4019	1.9241E-05	D - A; D - B; D - C
Uracil	27.248	4.1938E-06	5.3774	1.9942E-05	B - A; D - A; D - B; D - C
Oleic acid	26.499	4.9428E-06	5.306	2.3034E-05	A - B; A - C; A - D
4-Amino-4-deoxychorismate	25.609	6.0412E-06	5.2189	2.76E-05	A - B; A - D; C - B
D-erythro-3-Methylmalate	25.037	6.8931E-06	5.1616	3.0886E-05	A - D; B - D; C - D
Glucosamine	23.966	8.8889E-06	5.0511	3.9078E-05	A - B; A - C; A - D
Gamma-Aminobutyric acid	23.255	1.0577E-05	4.9756	4.5639E-05	B - A; D - A; D - B
dCMP	22.1	1.4162E-05	4.8489	5.9994E-05	C - A; C - B; C - D
L-Proline	21.054	1.8652E-05	4.7293	7.7606E-05	A - D; B - D; C - D
dTDP	20.584	2.1184E-05	4.674	8.6594E-05	A - C; B - C; D - C
Inositol 1,3,4,5,6-pentakisphosphate	20.449	2.1985E-05	4.6579	8.832E-05	A - B; A - C; A - D
Shikimate 3-phosphate	20.315	2.2808E-05	4.6419	9.0073E-05	A - B; A - D
Docosahexaenoic acid	20.147	2.3898E-05	4.6216	9.2803E-05	A - B; A - C; A - D
8,11,14-Eicosatrienoic acid	19.752	2.6697E-05	4.5735	0.00010091	A - B; A - C; A - D
Folic acid	19.731	2.6852E-05	4.571	0.00010091	C - A; C - D
5-Amino-6-ribitylamino uracil	18.937	3.374E-05	4.4719	0.00012478	A - D; C - B; C - D
Deoxycytidine	18.824	3.4877E-05	4.4575	0.00012697	C - A; C - B; C - D
S-Formylglutathione	17.104	5.8882E-05	4.23	0.00020953	A - B; A - D
Glyceric acid	17.079	5.9353E-05	4.2266	0.00020953	A - D; B - D; C - D
4a-Carbinolamine tetrahydrobiopterin	16.921	6.2419E-05	4.2047	0.00021707	D - A; D - B; D - C
4-Acetamidobutanoic acid	16.862	6.3596E-05	4.1966	0.00021791	C - B; D - B
N-Acetyl-L-glutamate 5-semialdehyde	16.579	6.9669E-05	4.157	0.00023526	A - B; C - B
Diacetyl	16.447	7.2713E-05	4.1384	0.00024203	B - A; D - A; D - C
Guanosine monophosphate	16.366	7.4655E-05	4.1269	0.00024499	B - C; D - C
Glycerophosphocholine	15.861	8.8228E-05	4.0544	0.00028552	A - D
2S-acetyl-2-hydroxy-butanoic acid	15.747	9.1688E-05	4.0377	0.00029265	B - D
Deoxyuridine	15.364	0.00010443	3.9812	0.00032881	A - D; B - D
S-Lactoylglutathione	14.859	0.00012447	3.9049	0.00038668	D - A; D - B; D - C
N4-Acetylaminobutanal	14.533	0.00013971	3.8548	0.00042581	B - A; D - A
Trypanothione disulfide	14.513	0.00014072	3.8517	0.00042581	D - A; D - C
Shikimic acid	14.303	0.00015177	3.8188	0.00045337	A - D
Phenylacetaldehyde	14.084	0.00016433	3.7843	0.00048466	D - A; D - B; D - C
Glucuronic acid	14.03	0.00016763	3.7757	0.00048822	A - B; A - C

Tetrahydrobiopterin	13.908	0.0001753	3.7562	0.00050427	D - A; D - C
Inosine	13.86	0.00017848	3.7484	0.00050715	A - D; C - D
Adenine	13.601	0.00019657	3.7065	0.00055182	D - A
Chitin	13.541	0.00020103	3.6967	0.00055763	D - A; D - B
L-Asparagine	13.388	0.00021303	3.6716	0.00058395	D - A; B - C; D - C
Nicotinic acid	13.347	0.00021633	3.6649	0.00058611	A - B
Putrescine	13.105	0.00023732	3.6247	0.00063559	C - A; C - B
Uridine 5'-monophosphate	12.873	0.00025969	3.5855	0.00068759	B - A; C - A
N-Acetyl-D-glucosamine	12.687	0.00027936	3.5538	0.00072589	B - A; D - A
5-Hydroxy-L-tryptophan	12.678	0.00028039	3.5522	0.00072589	C - A; C - B; C - D
Ornithine	12.604	0.00028872	3.5395	0.00073924	D - A; D - B
Mannitol	12.434	0.00030889	3.5102	0.0007802	D - A; D - B; D - C
Mevalonic acid-5P	12.414	0.00031141	3.5067	0.0007802	C - D
3-Oxo-OPC6-CoA	12.113	0.00035167	3.4539	0.00087011	A - B; A - C; A - D
5,10-Methylene-THF	12.092	0.00035477	3.4501	0.00087011	B - A; C - A; D - A
Stearic acid	11.99	0.0003698	3.432	0.00089755	A - C; D - C
N-Acetyl-D-Glucosamine 6-Phosphate	11.744	0.00040943	3.3878	0.00098347	B - D; C - D

Table S3. Correlation matrix between phenolic and flavonoid compounds in *P. columbinus* extracts and antioxidant effects in selected experimental models.

	DPPH Test	***ABTS Test***	**Linoleic Assay**	***Gallic acid***	***Hydroxy-tyrosol***	***Catechin***	***Chloro-genic acid***	***Epicate-chin***	***Benzoic acid***
DPPH Test	1								
ABTS Test	0,997	1							
Linoleic Assay	0,885	0,916	1						
Gallic acid	0,890	0,920	0,938	1					
Hydroxytyrosol	-0,073	0,002	0,305	0,385	1				
Catechin	-0,106	-0,029	0,360	0,282	0,898	1			
Chlorogenic acid	-0,083	-0,007	0,292	0,378	1,000	0,890	1		
Epicatechin	-0,707	-0,662	-0,465	-0,319	0,698	0,529	0,709	1	
Benzoic acid	0,780	0,740	0,557	0,423	-0,619	-0,466	-0,631	-0,994	1

Matrix analysis reveal a strong correlation (higher than 89%) of quantitative presence of gallic acid in the extracts and the antioxidant activities. On the other hands antioxidant properties of the extracts are less affected by the presence of other detected flavonoids and phenolics.
